# Identification of a Potential ISR Determinant from *Pseudomonas aeruginosa* PM12 against Fusarium Wilt in Tomato

**DOI:** 10.3389/fpls.2017.00848

**Published:** 2017-05-31

**Authors:** Sabin Fatima, Tehmina Anjum

**Affiliations:** Institute of Agricultural Sciences, University of the PunjabLahore, Pakistan

**Keywords:** Fusarium wilt, tomato, induced systemic resistance, *Pseudomonas*, 3-hydroxy-5-methoxy benzene methanol

## Abstract

Biocontrol of plant diseases through induction of systemic resistance is an environmental friendly substitute to chemicals in crop protection measures. Different biotic and abiotic elicitors can trigger the plant for induced resistance. Present study was designed to explore the potential of *Pseudomonas aeruginosa* PM12 in inducing systemic resistance in tomato against Fusarium wilt. Initially the bioactive compound, responsible for ISR, was separated and identified from extracellular filtrate of *P. aeruginosa* PM12. After that purification and characterization of the bacterial crude extracts was carried out through a series of organic solvents. The fractions exhibiting ISR activity were further divided into sub-fractions through column chromatography. Sub fraction showing maximum ISR activity was subjected to Gas chromatography/mass spectrometry for the identification of compounds. Analytical result showed three compounds in the ISR active sub-fraction viz: 3-hydroxy-5-methoxy benzene methanol (HMB), eugenol and tyrosine. Subsequent bioassays proved that HMB is the potential ISR determinant that significantly ameliorated Fusarium wilt of tomato when applied as soil drench method at the rate of 10 mM. In the next step of this study, GC-MS analysis was performed to detect changes induced in primary and secondary metabolites of tomato plants by the ISR determinant. Plants were treated with HMB and *Fusarium oxysporum* in different combinations showing intensive re- modulations in defense related pathways. This work concludes that HMB is the potential elicitor involved in dynamic reprogramming of plant pathways which functionally contributes in defense responses. Furthermore the use of biocontrol agents as natural enemies of soil borne pathogens besides enhancing production potential of crop can provide a complementary tactic for sustainable integrated pest management.

## Introduction

Tomato (*Solanum lycopersicum* L.) is a member of family Solanaceae, cultivated worldwide, ranked first among the processing crops and second as a vegetable crop. In Pakistan it is cultivated on about 58.196 thousand hectares with an annual production of 574.052 thousand tons (FAO, 2013). It contains valuable nutrients like vitamin A and C, calcium, phosphorus, potassium and magnesium (United States Department of Agriculture [USDA], 2009). It is also a source of an antioxidant compound named lycopene that has been found helpful against cancer (Miller et al., 2002). Tomato wilt caused by the fungus *Fusarium oxysporum* Schlecht. f. sp. *lycopersici* (Sacc.) W.C. Snyder et H.N. Hansen (FOL) is an alarming disease, causing yield losses up to 25% (Fravel et al., 2005; Park et al., 2013). Disease control strategies include use of resistant tomato varieties with cultural, chemical and biological control (Agrios, 2005; Pottorf, 2006). Cultural control lost its effectiveness as pathogen has a wide host range. Use of resistant varieties is futile due to chances of mutation in Fusarium spp. Chemical control is now losing its ground due to adverse effects of chemicals on environment and soil microbiota that calls for alternative inputs with lower dependency on chemicals for sustainable agriculture (Lucas, 2011). Recently used biological control employed induced systemic resistance (ISR) mechanism using rhizospheric plant growth-promoting bacteria against fungal pathogens (Pieterse et al., 2014).

Plants are devoid of an immune system which is the chief characteristic of mammals. To deal with different types of pathogens, plants have weakly inducible or constitutive defense systems including plant cell walls, cuticles, phytoanticipins (Underwood, 2012; Newman et al., 2013) and inducible defenses in which plants activate their immune system by the stimulus of signal molecules known as elicitors (Henry et al., 2012; Maffei et al., 2012; Newman et al., 2013). Elicitors can be derived from natural/living organisms like plants and microbes or generated synthetically (Walters et al., 2013).

Plant pathogens stimulate the host plant to activate defense responses against the invaders but this weak defense reaction will not limit the spread of the pathogen into the host plant (Thordal-Christensen, 2003). However, the defense responses can be enhanced by triggering the plant before pathogen attack. Interaction of some rhizobacteria with the plant roots has proven to increase plant resistance against some pathogenic bacteria, fungi and viruses. This phenomenon is termed as ISR (Lugtenberg and Kamilova, 2009). Application of microorganisms to control diseases, which is a form of biological control, is an environment-friendly approach (Lugtenberg and Kamilova, 2009). The direct mechanism of PGPR in biocontrol involves antagonism to soil borne pathogens (Supplementary Data Sheet 1) and indirect mechanism depends on induction of systemic resistance (Choudhary, 2011; Glick, 2012). Generally competition for nutrients, niche exclusion, ISR and antifungal metabolites production are some of the chief modes of biocontrol activity in PGPR (Lugtenberg and Kamilova, 2009). Many rhizobacteria have been reported to produce antifungal metabolites like hydrogen cyanide (HCN), phenazines, pyrrolnitrin, 2, 4-diacetylphloroglucinol, pyoluteorin, viscosinamide, and tensin (Bhattacharyya and Jha, 2012). In 2009, De Vleesschauwer and Höfte (2009) coined the term ISR for resistance induced by PGPR which was found to be independent of the pathway involved.

In case of microbially induced resistance the best option is the use of plant growth promoting rhizobacteria (PGPR) as potential elicitors of plant defense mechanisms (Liu et al., 1995). On the onset of plant colonization by the rhizobacteria, metabolic changes may occur in the host, i.e., production of phytoalexins (Van Peer et al., 1991), accumulation of pathogenesis related (PR) Proteins (Zdor and Anderson, 1992) or deposition of structural barriers, etc. (Benhamou et al., 1996a,b).

Origin of elicitor compounds may be biological (plant or microbe derived) or synthetic like beta-amino-butyric acid (BABA), *cis*-jasmone and acibenzolar-*S*-methyl (ASM) (Walters et al., 2013). Plants generally recognize three types of chemical elicitors viz; microbe-associated molecular patterns (MAMPs) derived from beneficial microbes, pathogen-associated molecular patterns (PAMPs) released by pathogenic microbes and damage-associated molecular patterns (DAMPs) produced by plants on injury by insects or herbivores or even during microbial degradation (Henry et al., 2012; Newman et al., 2013). These aforementioned molecules are called “patterns that elicit immunity” (PEIs) which are recognized by plants through transmembrane pattern recognition receptors (PRRs) (Jones and Dangl, 2006; Maffei et al., 2012; Newman et al., 2013). After recognition of MAMPs or DAMPs elicitors pattern-triggered immunity (PTI) is activated in plants. This stimulation of defense reaction restricts the pathogen making plant resistant to additional pathogen attack through the mechanism of induction of systemic resistance (Henry et al., 2012).

Bacteria belonging to the genus *Pseudomonas* involve pathogen inhibition via competition and/or antagonism (Haas and Défago, 2005) and by developing direct interactions with the host plants through ISR (Bakker et al., 2007). *Pseudomonas aeruginosa* PM12, used in this study was isolated from healthy tomato roots from vegetable garden of University of the Punjab, Lahore, Pakistan (Fatima and Anjum, 2016). It was characterized on molecular grounds through sequencing of 16S rRNA (900bp) and alignment at GeneBank (NCBI, MaryLand), allotted accession number KT966743 (Fatima and Anjum, 2017).

Induced systemic resistance is involved in synthesis of enhanced levels of various secondary metabolites engaged in plant defense mechanisms. These include phytoalexins that increase plant resistance through their toxic action against pathogens. Phytoalexins, i.e., sesquiterpenoids are synthesized by the members of Solanaceae and isoflavonoids are produced in individuals of the Papilionaceae. They inhibit germination of fungal spores and retard fungal growth. Plant tissues exhibiting ISR show increased activity of phenylalanine ammonia lyase (PAL) that leads to resistance response in plants (Verhagen et al., 2010).

Metabolomics is one of the most rapidly growing areas of contemporary science. This technology is now being used to route out reprograming and metabolic fluctuations in plant pathways (De Vos et al., 2007; Zhao et al., 2015). Current investigation revealed that compatible host pathogen interactions are characterized by a lower level of certain defense-related mechanisms compared with host pathogen interactions in the presence of bacterial ISR elicitor that leads to a more dynamic metabolic response over the course of colonization. Furthermore, our results demonstrate that elicitor (HMB) from *P. aeruginosa* (PM12) induces production of secondary metabolites involved in defense pathways of tested plant.

## Materials and Methods

### Fungal and Bacterial Strains

Virulent strain of *F. oxysporum* isolated from diseased tomato plants growing in vegetable garden of University of the Punjab, Lahore was cultured on potato dextrose agar (PDA difco) for 10 days. Conidia were harvested by gentle scraping in sterile water and pathogen inoculum was prepared by adjusting the concentration to 10^5^conidia/ml using haemocytometer. *P. aeruginosa* (PM12) was grown on LB broth medium (100 ml) for 24 h at 35°C. For collecting extracellular metabolites culture was pelleted by centrifugation at 4000 *g* for 15 min, and the supernatant obtained was processed for ISR assay. Intracellular metabolites were extracted using sonication. Bacterial cell lysis was performed six times through sonication at resonance amplitude for 15 s at 4°C.

### Preliminary Screening of ISR Determinants from *P. aeruginosa* PM12

This study was performed to identify the involvement of intracellular metabolites and cell-free culture filtrates (CFCF) of *P. aeruginosa* for inducing systemic resistance against fungal wilt in tomato. Two-weeks old seedlings of Fusarium wilt susceptible tomato variety “Rio-Grande” seeded in sterilized pot media. In total, 50 ml of both extracts (intracellular and extracellular) were supplied to the allocated plastic pots containing 0.5 kg sterilized soil and after a period of 3 days these pots were inoculated with 50 ml of pathogen @ 10^5^conidia/ml. Positive control consisted of *P. aeruginosa* PM12 suspension made in water by adjusting the concentration to 10^4^ cfu/ml. Fifty milli liter of sterile distilled water was provided to the pots designated as untreated control. All the pots were incubated for 14 days under greenhouse conditions. There were ten replicates against each treatment and experiment was conducted twice. To determine the disease index (DI), wilting was scored based on the criteria developed by Epp (1987) (0 = no wilt symptoms; 1 = less than 25% of the plant turned yellow; 2 = yellowing and browning covered nearly 50% of plant; 3 = whole plant turned brown and died). The equation described by Cachinero et al., 2002 was used to calculate the DI.

DI = [(Σ ni × si)/(N × S)] × 100

where, ni = the number of tomato plants with wilt symptoms, si = value of the symptom score, N = the total number of tested plants, and S = the highest value of the symptom score.

### Isolation of Bioactive Compound(s) from Extracellular Metabolites of *P. aeruginosa* (PM12)

Methodology proposed by Sumayo et al. (2013) was used to isolate bioactive compound (s) from extracellular metabolites of *P. aeruginosa* (PM12). Extracellular metabolites of *P. aeruginosa* PM12 were obtained as described previously and extracted twice with double volumes of organic solvents such as ethyl acetate, chloroform, n-hexane and n butanol. All the organic extracts were dried in a rotary evaporator at 50°C, mixed in 10% DMSO and subjected to ISR experimentation.

#### Primary Screening of Bacterial Metabolites Extracted with Various Solvents for ISR Activity

Seeds of the variety “Rio-Grande” were sown in sterilized media and after 2 weeks of emergence seedlings were transplanted in plastic pots (4 inches diameter) containing sterilized sandy loam soil. Pots were subjected to pathogen inoculum and bacterial metabolites extracted with various organic solvents for each solvent separately. After 1 week of incubation data regarding the disease development was recorded and the solvent treatment found most suitable for the disease reduction was further screened for ISR determinant/s.

### Column Chromatography of the Active Metabolites and Selection of Sub Fraction Involved in ISR

Bacterial metabolite exhibiting highest ISR activity was further fractioned through silica gel column chromatography. Column was washed with double volumes of methanol and ethyl acetate. The extract was fractioned using stepwise elution method with increased concentration of methanol in ethyl acetate. Obtained eluates were evaporated and mixed in 10% DMSO and checked for ISR activity in a test tube assay. Negative control received pathogen inoculum with 10% DMSO. Seeds of the variety “Rio-Grande” were surface sterilized with 1% NaOCl and sown in culture tubes containing Murashige and Skoog (MS) medium. Tubes were incubated in a growth chamber at 25°C for seedling development. Seedlings were then provided with different bioactive fractions for ISR activity separately. After 2 days of seedlings treatment, 10 μl of pathogen inoculum was provided to each tube @ 10^5^condia/ml. After 1 week of incubation data was recorded regarding DI. Experiment was repeated twice and there were ten replicates for each treatment.

### Identification of ISR Determinant(s) by GC-MS Analysis

Induced systemic resistance active sub-fraction obtained from silica gel chromatography was further subjected to instrumental analysis for identification of potential ISR determinant/s. This study was executed in Agilent GC/MS apparatus including capillary column (0.25 ID × 30 M × 0.25 μM film thicknesses) in which electron ionization was used as ion source. Helium with flow rate of 1.0 ml/min was supplied as carrier gas. Column temperature of apparatus was maintained at 30°C for 3 min which was increased at 50°C/min to 180°C and by 40°C/min to 200°C.

### ISR Bioassay with Pure Compounds

Another independent experiment was performed to screen ISR active biochemicals from *P. aeruginosa*. Biochemicals found in ISR active sub-fraction were purchased from the market. Three concentrations, i.e., 0.1, 1.0 and 10 mM of each of the pure biochemicals were applied as soil drench method, application of inoculum (bacterial/pathogen origin) to the soil surrounding the roots of transplanted seedlings of tomatoes, at the rate of 50 ml/pot (Algam et al., 2005). Data regarding DI was taken as described earlier after 15 days of treatment applications.

### Analysis of Metabolite Profile of Tomato Plants in the Presence of ISR Elicitor from *P. aeruginosa* PM12

It was found in the previous experiment that HMB acted as the most active compound involved in elicitation of ISR in tested tomato plants therefore another independent experiment was carried out to study metabolic transitions brought about in the tomato plants under the influence of HMB elicitor.

#### Plant Growth and Inoculation

Seedlings of the tomato variety ‘Rio-Grande’ were raised in sterilized sandy loam soil media. After 3 weeks of emergence seedlings were transplanted @ 1 seedling/ pot in plastic pots (4 inches diameter) containing sterilized sandy loam soil. Four treatments were made in this experiment viz; T1 = Plants receiving ISR elicitor (HMB) + FOL, T2 = Plant receiving ISR elicitor (HMB) alone, T3 = Plant receiving FOL to serve as pathogen control, T4 = Plants receiving distilled sterilized water to serve as untreated control.

After 3 days of seedling transplantation, 50 ml of 10 mM ISR elicitor (Pure compound) was supplied to the tomato plants. After 2 days of treatment, FOL inoculum was provided @ 50 ml of conidial suspension (see Fungal and Bacterial Strains). Plants were incubated under greenhouse conditions. Experiment was conducted twice having 15 plants in each replicate.

#### Extraction of Plant Metabolites

After 1 week of pathogen challenge to the seedlings plant metabolites were extracted. Leaf samples (1 g) from young shoots were taken from treated and untreated plants separately. Samples were crushed in liquid nitrogen and the resulting powder was dissolved in 10 ml of extraction solution comprising of methanol, chloroform and water mixed in a ratio of 80:10:10, respectively. Dissolved material was kept overnight at room temperature. Afterward metabolite extraction was done using micro filters.

##### Derivatization

Derivatization transforms a chemical compound into its derivative having similar chemical structure suitable for GC-MS analysis. Plant extract (0.3 ml) was mixed with 0.1 ml of ribitol (internal standard) inside a glass container. It was dried using N_2_ gas. After drying added 25 μl of methoxyamine hydrochloride (MOX), mixed it and left overnight at room temperature. Afterward, 80 μl of *N*-methyl-*N* trimethylsilyl- trifluoroacetamide (MSTFA) was supplied to the solution and left for 2 h at room temperature (Warth et al., 2014).

##### GCMS Analysis of Derivatized Plant Metabolites

Gas chromatography-mass spectrometry set up consisted of capillary column (0.25 ID × 30 m × 0.25 μm film thicknesses) and electron ionization was used as an ion source. Helium was used as a carrier gas at the flow rate of 1.0 ml/min. Temperature of the capillary column was adjusted at 30°C for 3 min which was raised to 50°C /min to 180°C and by 40°C/min to 200°C / min. Derivatized plant extract was injected in GC-MS machine @ 1 μl.

##### Plant Metabolites Analysis

Metabolite analysis of tomato was performed using method devised by Lisec et al. (2006). Metabolite identification was carried out by comparing spectrum with NIST library. Levels of different metabolites were determined through Mzmine software package^1^; values obtained were log2-transformed (Steinfath et al., 2008) and normalized to show identical medium peak sizes per sample group. ClustVis^2^ online tool was used to make heatmap.

### Statistical Analysis

The data was analyzed by performing ANOVA, and the significance of difference between the treatments was determined by DNMRT at *P* < 0.05 using software DSASTAT (Onofri, 2007).

## Results

### Initial Screening of Metabolites Involved in ISR from *Pseudomonas aeruginosa* PM12

**FIGURE 1:**
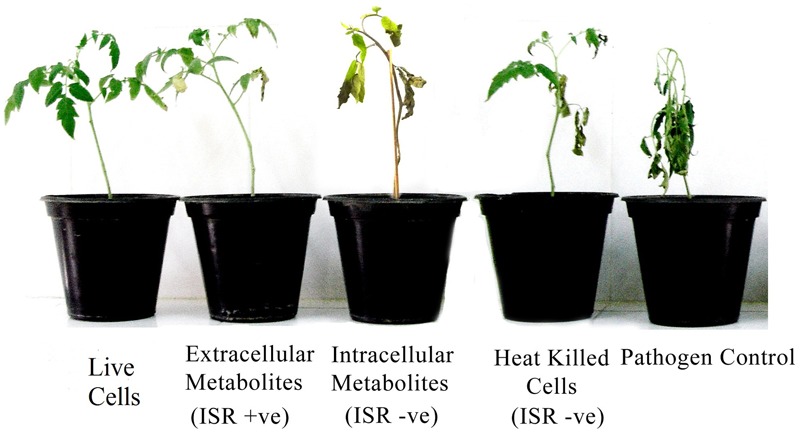
**Disease severity of tomato plants treated with *Pseudomonas aeruginosa* PM12 (live cells and heat treated cells), as well as with the intracellular and extracellular metabolites followed by *Fusarium oxysporum* challenge.** Tomato plants shown are from 2 weeks of post-inoculation.

Extracellular metabolites of *P. aeruginosa* (PM12) were found to be actively involved in suppressing Fusarium wilt of tomato under greenhouse conditions (**Table 1**). Significant reductions in the DI up to 73% were recorded in case of the extracellular metabolites that was almost equal to the efficacy of live cells, i.e., 75.86%. Intracellular metabolites were not found capable in suppressing the disease to a significant level. Resultantly, it was concluded that extracellular metabolites are involved in disease suppression and contains ISR determinant/s (**Figure 1**).

**Table 1:** Preliminary screening of ISR determinant from *P. aeruginosa* PM12.

Treatments	Disease index	Biocontrol effect
Live cells	20.54^D^ (± 1.82)	71.17^A^ (± 05.21)
Heat killed cells	80.51^B^ (± 6.54)	11.67^C^ (± 01.27)
Intra-cellular components	79.23^B^ (± 04.97)	20.13^B^ (± 01.88)
Extracellular metabolites	22.88^C^ (± 01.66)	70.12^A^ (± 06.47)
Pathogen control	85.08^A^ (± 0.792)	–

*Capital letters represents levels of significance as governed by ANOVA and DNMRT at (*P* > 0.05). Values with ± signs represent standard error between different replicates of same treatment.*

### Searching of ISR Determinants from Extracellular Metabolites of Bacterial Strains

**FIGURE 2:**
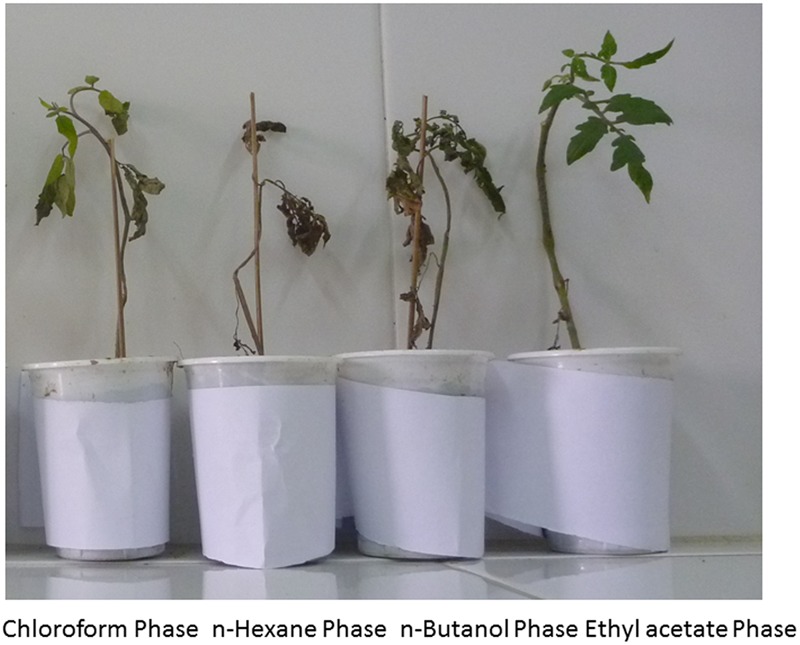
**Protective effect against Fusarium wilt symptoms of four solvent fractions from the extracellular metabolites secreted by *P. aeruginosa* (PM12)**.

In this experiment extracellular metabolites/CFCF of *P. aeruginosa* PM12 were examined for the presence of ISR determinant/s. Extraction of extracellular metabolites was done by using different organic solvents and then applied to the plants as soil drench method and was challenged with the pathogen afterward. Compound/s present in ethyl acetate fraction of extracellular metabolites significantly reduced wilt disease in tomato plants when observed on visual grounds (**Figure 2**).

### Identification of ISR Determinant by GC/MS Analysis

**FIGURE 3:**
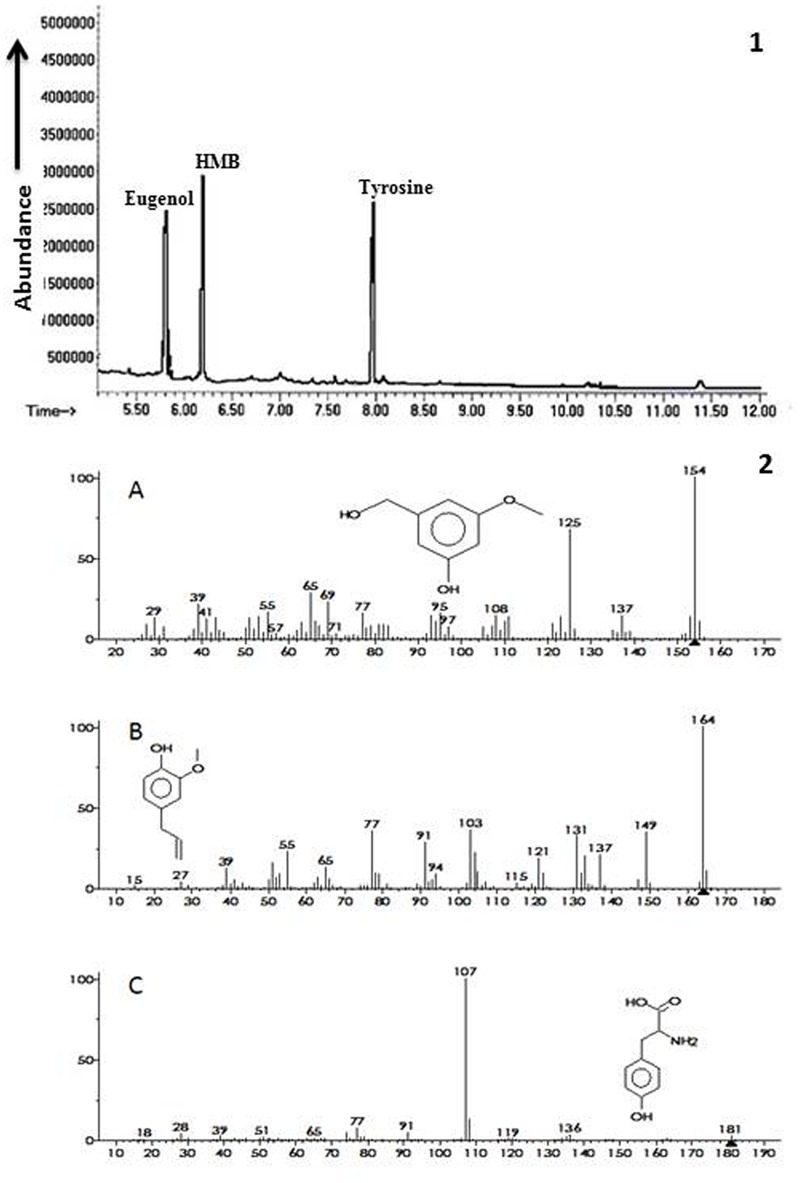
**Biochemicals observed in ISR active sub fraction found in extracellular metabolites of *P. aeruginosa* (PM12).** 1 = Chromatogram obtained from Gas Chromatography and Mass Spectrometry (GC/MS) analysis for identification of ISR determinants. 2 = Ionization patterns of A = 3-hydroxy-5-methoxy benzene methanol (HMB), B = Eugenol, C = Tyrosine.

Ethyl acetate fraction of extracellular metabolites exhibiting maximum ISR activity was further processed via column chromatography using step wise elution method and ISR experiment was performed in a test tube assay. Sub-fraction showing maximum disease suppression was then analyzed through GC/MS. Chromatogram obtained from GC/MS analyses showed three peaks that were identified as 3-hydroxy-5-methoxy benzene methanol (HMB), eugenol and tyrosine, respectively (**Figure 3**).

**FIGURE 4:**
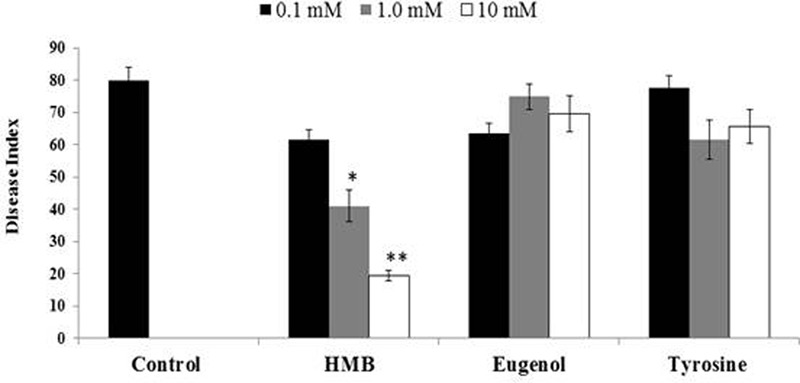
**Influence of soil drench application of pure biochemicals present in ISR active sub-fraction on the disease development on tomato plants after inoculation with Fusarium wilt pathogen.** HMB = 3-hydroxy-5-methoxy benzene methanol. Vertical bars represents standard errors. Asterisks indicate statistically significant reduction in disease index as compared to pathogen control as governed by ANOVA at (*P* < 0.05).

Biochemicals obtained were again tested for ISR activity in their pure form. The ISR bioassay showed that only HMB significantly (*P* ≤ 0.05) reduced disease severity to 76.25% at 10 mM concentration. Whereas eugenol and tyrosine were not found efficient in reducing disease severity at the same concentration (**Figure 4**).

### Metablomic Analysis of Tomato Plants

**FIGURE 5:**
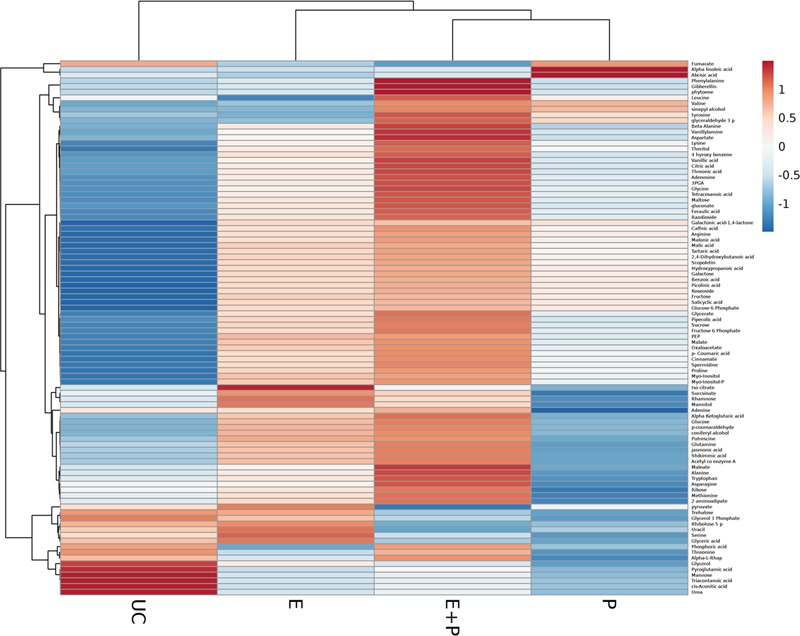
**Cluster of differentially produced metabolites obtained by treating tomato plants with ISR elicitor and pathogen.** Each row represents a different abundance of metabolite, while each column represents a sample. The colors in the heat map depicts the intensity of log2- fold change in metabolite levels. E+P = Plants receiving ISR elicitor + Pathogen, E = Plant receiving ISR elicitor, P = Pathogen control, UC = Non-treated control.

This study explored metabolic transitions observed in the tomato variety “Rio-Grande” under the stimulus of ISR elicitor (HMB) using GC/MS analysis. Whole metabolome of plants inoculated with HMB and pathogen in either combination were studied to elucidate ISR mechanism. In four different treatments, 94 metabolites were detected some of which were up regulated and some were down regulated as shown by different color schemes in heat map (**Figure 5**). Metabolite values obtained using Mzmine software were log2-transformed (Steinfath et al., 2008). Heat map was generated using online ClustVis tool with row wise scaling and correlation-based clustering. Most pronounced changes were depicted in metabolite levels of the plants receiving elicitor + pathogen making plants more resistant against pathogen attack by timely accumulation of defense related chemicals (**Figure 5**). Changes brought about in central metabolites under the stimulus of different treatments were normalized to respective control and were expressed in fold change values. Most metabolic fluctuations were documented in primary and secondary metabolism, signaling and defense pathways. Treatment receiving elicitor and pathogen showed variation in primary metabolism with different intensities. Upregulation of various metabolites involved in phenylpropanoid pathway was documented in case of the elicitor, e.g., 4-hydroxybenzene, cinnamate and tryptophan were increased to 3.19-, 2.31-, and 1.24-folds, respectively (**Figure 5**). Similarly plants treated with elicitor and pathogen significantly enhanced levels of most of the metabolites taking part in amino acid metabolism and TCA pathway (**Figure 5**). These findings illustrate that synergistic effect of the elicitor and the pathogen in inducing systemic resistance was more pronounced due to the active participation of the pathogen in some cases or elicitor in other.

Plants treated with the elicitor (HMB) showed enhanced levels of fructose, glucose and sucrose to 3.4-, 3.1-, and 1.2-folds, respectively. Whereas concentration of trehalose was found to be 0.9-fold less as compared to the untreated control (**Figure 5**).

In case of phosphorylated metabolites there was an increasing trend in the concentration of fructose-6- phosphate, glucose-6-phosphate and myo-inositol-phosphate to 3.9-, 3.3-, and 1.9-folds, respectively. Whereas concentration of glycerol-3-phosphate (0.9-fold) was decreased to 0.9-fold in the presence of the elicitor in comparison to the untreated control.

Plants treated with the elicitor showed increased levels of organic acids including α ketoglutarate (2.7-fold), malate (2.8-fold), oxaloacetate (2.3-fold), citrate (2.1-fold) and succinate (1.4-fold). Whereas low levels of fumarate (0.4-fold) and *cis*- aconitate (0.3-fold) were recorded in comparison to the untreated control.

Elicitor treated plants also showed raised quantities of polyamines like picolinic acid (7.7-fold), pipecolic acid (4.7-fold), putrescine (2.8-fold), and spermidine (1.4-fold).

Amino acids like arginine, glutamine and proline levels were significantly increased to 3.04, 2.7 and 2.32-folds in case of the elicitor whereas leucine, tyrosine and threonine were decreased in comparison to the untreated control (**Figure 5**). Methionine acts as a precursor for ethylene biosynthesis through *S*-adenosyl methionine (SAM) (Bleecker and Kende, 2000). Methionine levels were significantly raised in plants under the influence of elicitor and elicitor + pathogen treatments.

**FIGURE 6:**
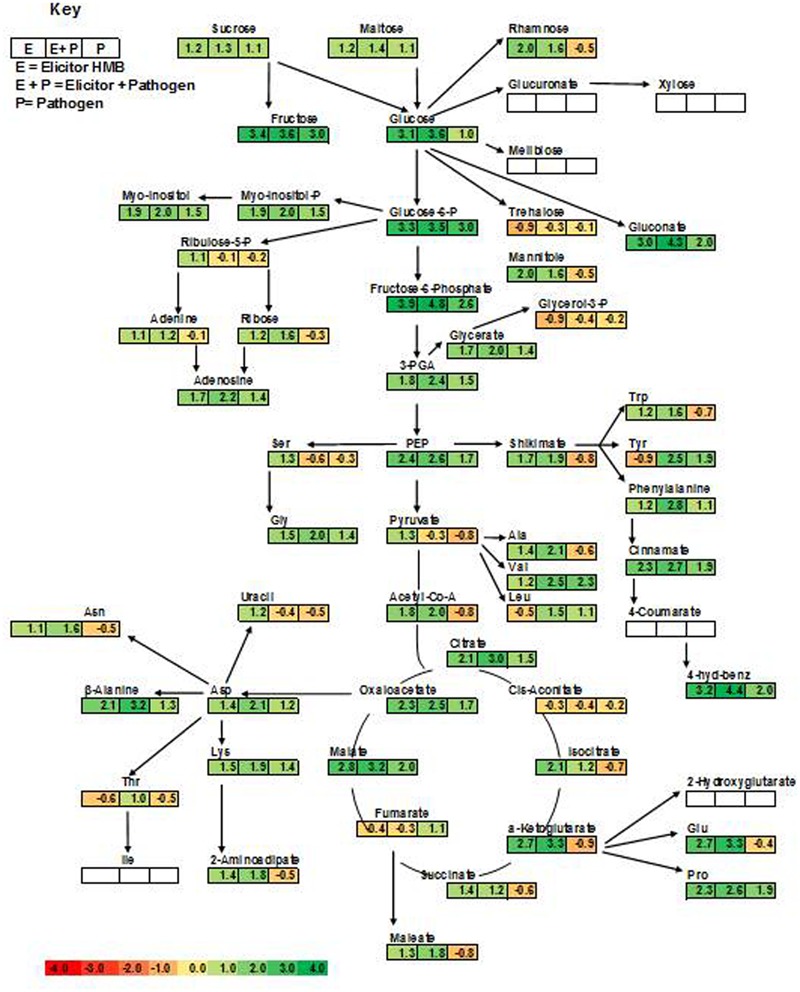
**Integration of the changes in the metabolites associated with selected metabolic pathways in the pathogen and elicitor-inoculated tomato plants at different combinations.** The metabolites identified from the primary and secondary metabolic pathways are shown. Each square represents the log2 ratios of the abundance after 7 days of treatment applications. Fold values of all the treatments as compared to untreated control were used. Metabolites in green color show significant increase over control as governed by ANOVA at *P* < 0.05. Red boxes represent no significant change as compared to control. White color represents metabolites that were not detected.

**FIGURE 7:**
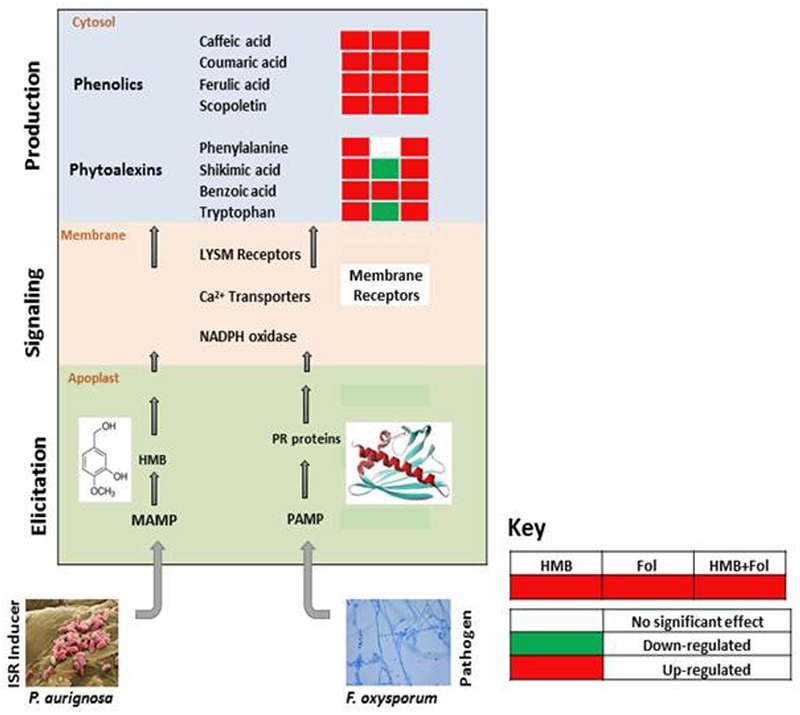
**Re-modulations in ISR signaling pathway of tomato plants induced by ISR elicitor (HMB) and Fusarium wilt pathogen in either combination**.

**FIGURE 8:**
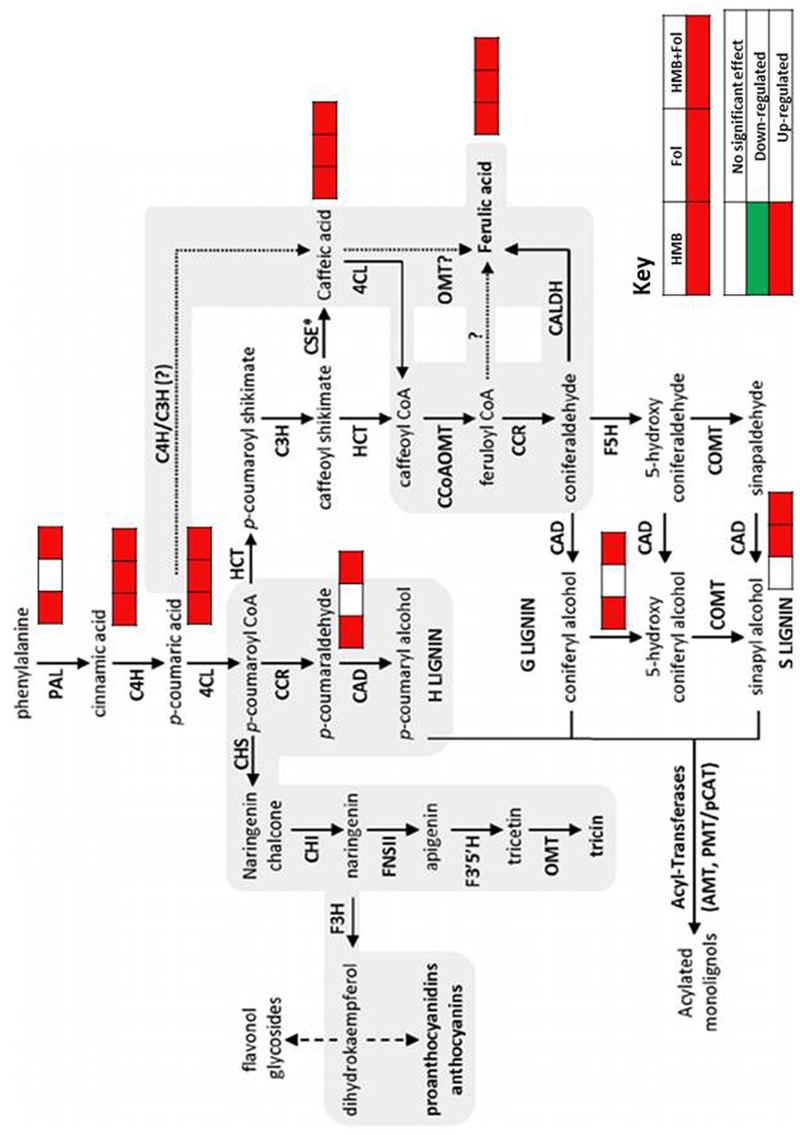
**Dynamics observed in phenylpropanoid pathway of tomato plants treated with ISR elicitor and *F. oxysporum* in either combination**.

Levels of salicylic acid were significantly upregulated in all the treatments in comparison to the untreated control. Plants treated with the elicitor and the pathogen showed increased levels of phenylalanine to 2.8-folds whereas application of the elicitor resulted in an increase of 1.2-folds as compared to the non-treated control. Metabolic pathways were drawn to show transitions in various metabolites in the presence of elicitor HMB (**Figures 6–8**). Metabolites belonging to the phenylpropanoid, glycolysis, photosynthesis and signal transduction pathways were significantly increased by the elicitor (HMB) in treated tomato plants.

Alterations in the metabolites of the plants receiving elicitor and elicitor + pathogen revealed that HMB elicitor from *P. aeruginosa* is responsible for inducing systemic resistance against Fusarium wilt in tomato.

## Discussion

### Isolating ISR Determinant(s) from *Pseudomonas aeruginosa* PM12

Recent attempts in exploiting non-pathogenic rhizospheric microbes in biocontrol programs have shown their involvement as antagonists and plant defense stimulators. However, the exact nature of biochemicals imparting systemic resistance in plants against diseases remains poorly understood. This research was performed to isolate potential ISR determinant/s from *P. aeruginosa* (PM12) against *F. oxysporum* f. sp. *lycopersici* in tomato plant.

*Pseudomonas aeruginosa* PM12 is a non-pathogenic strain that was isolated from tomato rhizosphere. To avoid the use of whole bacterium as it is categorized as opportunistic pathogen we focused on the signal compound/s involved in eliciting defense mechanisms of the plant and identified the ISR determinant from extracellular metabolites of *P. aeruginosa* PM12. Moreover opportunistic pathogens are microbes that are capable of causing disease only in people who are immunocompromised or are otherwise especially susceptible. A critical issue concerns the pathogenic strains proposed for registration as biopesticides, since these strains are typically isolated from the environment, for example agricultural fields, rather than as clinical specimens. As such, these strains have no history of actually causing disease and may not be able to do so (GPO, 1999).

Extracellular metabolites and live cells of *P. aeruginosa* PM12 showed significant (*P* ≤ 0.05) protection against Fusarium wilt whereas the intracellular metabolites were unable to provide any considerable control (**Table 1**). The ethyl acetate fraction of extracellular metabolites of the bacterium exhibited maximum ISR activity and was sub fractioned using column chromatography. The active sub fraction was then analyzed through GC/MS. The chromatogram obtained identified three compounds, i.e., HMB, tyrosine and eugenol. Reconfirmation of active biochemical was achieved using pure compounds which revealed HMB as the most capable component in mitigating Fusarium wilt in tomato.

The elicitor compound, HMB, isolated from *P. aeruginosa* (PM12), is not yet reported for its biological activity against Fusarium wilt so this is the first finding describing its involvement in systemic resistance in tomato. Colonization of plant roots with *P. fluorescens* enhanced plant defenses against pathogen by sensitizing the plant through signal compounds (Verhagen et al., 2004). Literature showed the use of benzene in making pesticides, detergents, drugs, dyes, rubbers, explosives and lubricants (Ceresana, 2014). Furthermore antifungal and antimicrobial activity of poly substituted benzene derivatives has been reported. The substituent attached to the benzene ring and its position affects the activity of the compound against pathogen (Uyanik et al., 2009). Similarly elicitor benzothiazole isolated from *Pseudomonas* sp. has shown inhibition of mycelial growth and germination from ascospores and sclerotia of *Sclerotinia sclerotiorum* (Fernando et al., 2005). Aldehydes, ketones and benzenes from *Bacillus amyloliquefaciens* have been known to restrict mycelial growth and spore germination of *F. oxysporum* (Yuan et al., 2012). Bacterial volatile compounds help in maintaining plant health through ISR and production of antifungal compounds (Weisskopf, 2013). As benzene is involved in protection of plants against different pests so it is concluded that benzene is the actual molecule in elicitor compound HMB for imparting resistance against FOL in tomato.

A considerable diversity of the elicitors involved in ISR has been observed in case of Pseudomonads. They include the building blocks of bacterial cells as well as extracellular compounds synthesized by the microbes (Jankiewicz and Kol-tonowicz, 2012). In current investigation HMB was discovered as an ISR determinant from *P. aeruginosa* PM12. Nowadays awareness regarding concept of immunity development in plants by the use of beneficial microbes is gaining momentum. In conventional agriculture system the identification of MAMPs from beneficial microbes and their use in formulation stimulates resistance in plants against pest attack.

Use of biocontrol agents in elicitation of resistance in plants is gaining importance these days. Some strains of *Pseudomonas* are clearly potent inducers of systemic resistance in plants (Van Wees et al., 1999; Meena et al., 2000).

### Mechanism behind Systemic Resistance Induced by *Pseudomonas aeruginosa* PM12 in Tomato Plants

Biological inducers have been known to elicit metabolites involved in different pathways like pentose pathway, signaling transduction and tricarboxylic acid cyclic, etc. (Verhagen et al., 2004; Shoresh and Harman, 2008; Damodaran et al., 2010; Brotman et al., 2012). In second phase of this study heat map was developed to show variations in metabolites under the influence of HMB and accentuated in physiological pathways. In this investigation upregulation of metabolites involved in phenylpropanoid pathway may lead to resistance development in tomato plants treated with the elicitor HMB.

A significant decrease in fumaric acid was recorded in the elicitor and elicitor + pathogen treated plants. These results are in connection with the findings of Doornbos et al. (2009) who inoculated Arabidopsis plants with the PGP and ISR-inducing bacterium WCS358r and observed decrease in intermediates of Krebs cycle that showed an increased demand for carbon respiration.

Brotman et al. (2012) reported that putrescine induction by *Trichoderma* spp. was related to growth promotion in *Arabidopsis thaliana*. Polyamines were also involved in various defense responses (Tonon et al., 2004) similarly in this study upregulation of putrescine was noted.

In plant microbe interactions, sugars serve as signaling molecules for resistance induction against the pathogens and this resistance is termed as “sweet immunity” (Bolouri Moghaddam and Van den Ende, 2013). Like sucrose is involved in activating plant immune responses against pathogens (Tauzin and Giardina, 2014). Sucrose is known to trigger the production of isoflavonoids against *F. oxysporum* in lupine (Morkunas et al., 2011). Trehalose stimulates the activity of phenyl ammonia lyase and peroxidase against powdery mildew in wheat (Reignault et al., 2001). Fructose, sucrose and glucose elicited PR-protein transcripts in tobacco in a SA-independent pathway (Herbers et al., 1996). Moreover Kawano and Shimamoto (2013) and Yamaguchi et al. (2013) found that chitin is responsible for stimulation of Mitogen-Activated Protein Kinase (MAPK) cascade, lignin and phytoalexins production in rice. In this study significant increase in quantities of some carbohydrates like fructose, glucose, mannose, etc., were also documented that are reportedly involve in plant growth and development.

Salicylic acid is involved in activation of defense responses in plants after challenging with a pathogen (Klessig et al., 2000). In a study enhanced levels of salicylic acid were documented when plants were challenged with pathogen or with *P. fluorescens* (Kumar and Sharma, 2013). Our results also showed upregulation of salicylic acid in the presence of the bacterial elicitor HMB.

Glutamate levels were significantly raised in treatments receiving the elicitor or elicitor and pathogen in combination. These results are in line with findings of Brotman et al. (2012). Glutamate acts as signaling compound in nitrogen signaling pathway (Forde and Lea, 2007).

Biochemicals like polyamines are involved in different physiological processes of the plants (Tonon et al., 2004). In this study plants primed with the elicitor (HMB) showed significant increase in polyamine levels. Likewise an increasing trend was also observed in organic acids in the HMB + pathogen treated plants. Shikimate pathway is stimulated by the synthesis of aromatic compounds which is responsible for defense response of plants (Tzin and Galili, 2010). Moreover shikimic acid concentration was also found to be enhanced under the effect of the bacterial elicitor HMB. Shikimic acid and malonic acid pathways are involved in phenolics synthesis leading to plant defense against pathogens. Phenolics include a variety of defense related compounds like anthocyanins, phytoalexins, flavonoids, furanocoumarins tannins and lignin (Freeman and Beattie, 2008). Present study illustrated the significance of metabolic dynamics engaged in defense mechanisms of the tomato plants infected with *F. oxysporum* ensured triggering of ISR by the bacterial elicitor HMB.

Phenylpropanoids like lignin, phytoalexins, flavonoids, tannins, etc., are the secondary metabolites of the plants produced under stress conditions (Vogt, 2010). They provide resistance in plants against phytopathogens through accumulation of lignin that acts as a barrier for penetration in plant tissues. Enzymes involved in phenylpropanoid pathways viz; PPO, PO and PAL impart rigidity and mechanical strength to the plant cell walls through production of lignin metabolites that act as barrier for invading fungal pathogens (Mohammadi and Kazemi, 2002; Anjum et al., 2012). Metabolic dynamics engaged in defense mechanisms of the tomato plants infected with *F. oxysporum* ensured elicitation of ISR by the bacterial elicitor HMB. Enhanced metabolite upregulation under influence of the elicitor + pathogen treatment may be due to the synergistic effect of ISR and SAR that may lead to better cope with the stress condition of the plant by activation of defense machinery.

## Conclusion

Findings of our study revealed that priming of plant roots with *P. aeruginosa* helps in suppressing Fusarium wilt by releasing an ISR elicitor HMB. Furthermore this elicitor compound is engaged in elicitation of defense mechanisms in tomato plants through metabolic transitions that leads to resistance induction against Fusarium wilt pathogen. Research regarding targeting of genes controlling the regulation of this elicitor compound will undoubtedly help in pest management practices in a sustainable manner.

## Author Contributions

SF and TA designed the study. TA revised the manuscript critically. All authors read and approved the final version of the manuscript.

## Conflict of Interest Statement

The authors declare that the research was conducted in the absence of any commercial or financial relationships that could be construed as a potential conflict of interest.

## Footnotes

1 http://mzmine.sourceforge.net/

2 http://biit.cs.ut.ee/clustvis/
