# Supplementary material for: Identification of a Potential ISR Determinant from Pseudomonas aeruginosa PM12 against Fusarium Wilt in Tomato
Source: Front Plant Sci. 2017 May 31;8:848. doi: 10.3389/fpls.2017.00848 (PMC5450013; doi:10.3389/fpls.2017.00848)
Supplement: Supplementary file 1 [file Data_Sheet_1.DOCX]

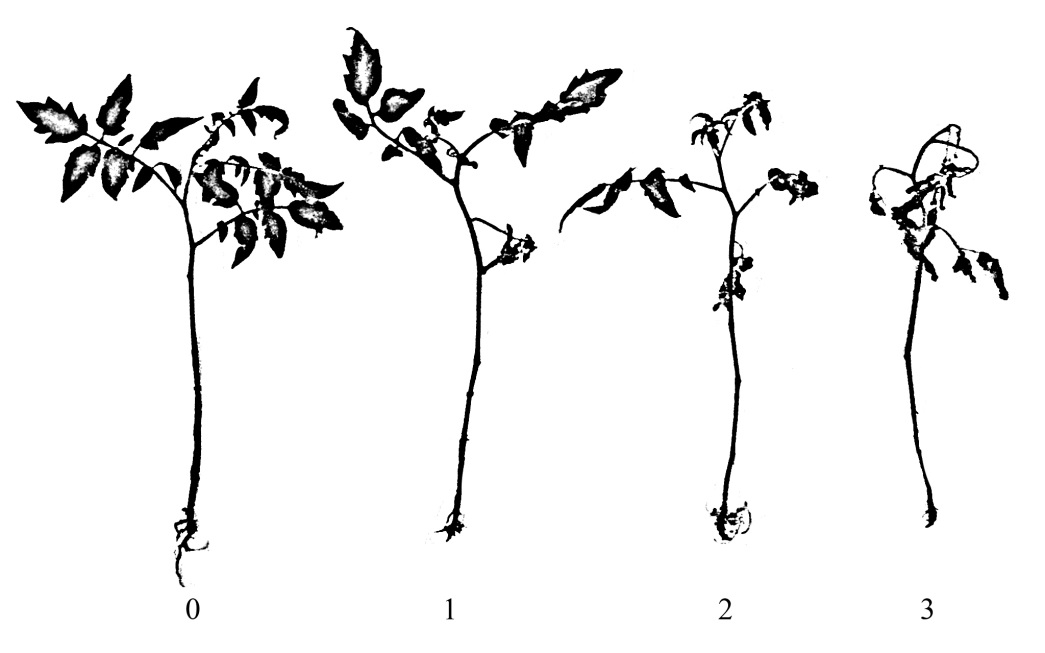


Wilting was scored based on the criteria developed by Epp (1987) (**0** = no wilt symptoms; **1** = less than 25% of the plant turned yellow; **2** = yellowing and browning covered nearly 50% of plant; **3** = whole plant turned brown and died). The equation described by Cachinero et al. 2002 was used to calculate the DI.

DI = [(Σni × si)/(N × S)] × 100

where, ni = the number of tomato plants with wilt symptoms, si = value of the symptom score, N = the total number of tested plants, and S = the highest value of the symptom score.
